# Growth Inhibition of *Beauveria bassiana* by Bacteria Isolated from the Cuticular Surface of the Corn Leafhopper, *Dalbulus maidis* and the Planthopper, *Delphacodes kuscheli,* Two Important Vectors of Maize Pathogens

**DOI:** 10.1673/031.011.0129

**Published:** 2011-03-10

**Authors:** A.V. Toledo, A.M. Alippi, A.M.M. de Remes Lenicov

**Affiliations:** ^1^ Centro de Investigaciones de Fitopatología (CIDEFl), Facultad de Ciencias Agrarias y Forestales, Universidad Nacional de La Plata, Calle 60 y 119, s/n, 1900, La Plata, Buenos Aires, Argentina; ^2^ División Entomología, Facultad de Ciencias Naturales y Museo, Universidad Nacional de La Plata, Paseo del Bosque s/n, 1900, La Plata, Buenos Aires, Argentina

**Keywords:** *Bacillus licheniformis*, *Bacillus megaterium*, *Bacillus mycoides*, *Bacillus pumilus*, *Bacillus subtilis*, *Bacillus thuringiensis*, bacterial antagonists, cicedellids, delphacids, entomopathogenic fungus

## Abstract

The phytosanitary importance of the corn leafhopper, *Dalbulus maidis* (De Long and Wolcott) (Hemiptera: Cicadellidae) and the planthopper, *Delphacodes kuscheli* Fennah (Hemiptera: Delphacidae) lies in their ability to transmit phloem-associated plant pathogens, mainly viruses and mollicutes, and to cause considerable mechanical damage to corn plants during feeding and oviposition. Fungi, particularly some members of the Ascomycota, are likely candidates for biocontrol agents against these insect pests, but several studies revealed their failure to invade the insect cuticle possibly because of the presence of inhibitory compounds such as phenols, quinones, and lipids and also by the antibiosis effect of the microbiota living on the cuticular surface of the host. The present work aims to understand interactions between the entomopathogenic fungus *Beauveria bassiana* (Balsamao-Crivelli) Vuillemin (Hypocreales: Cordycipitaceae) and bacterial antagonists isolated from the cuticular surface of *D. maidis* and *D. kuscheli.* A total of 155 bacterial isolates were recovered from the insect's cuticle and tested against *B. bassiana.* Ninety-one out of 155 strains inhibited the growth of *B. bassiana.* Bacterial strains isolated from *D. maidis* were significantly more antagonistic against *B. bassiana* than those isolates from *D. kuscheli.* Among the most effective antagonistic strains, six isolates of *Bacillus thuringiensis* Berliner (Bacillales: Bacillaeae (after *B. subtilis*)), one isolate of *B. mycoides* Flügge, eight isolates of *B. megaterium* de Bary, five isolates of *B.pumilus* Meyer and Gottheil, one isolate of *B. licheniformis* (Weigmann) Chester, and four isolates of *B. subtilis* (Ehrenberg) Cohn were identified.

## Introduction

Argentina is a leading maize-producing country, with an annual production of 15,500,000 tons. Several auchenorrynchan (Hemiptera: Auchenorryncha) species belonging to the Cicadellidae (leafhoppers) or the Delphacidae (planthoppers) families can reduce the yield and quality of maize grains because they transmit different plant pathogens, mainly viruses and mollicutes, and cause considerable mechanical damages during feeding and oviposition (Nault and Ammar 1989). The mentioned families include the largest number of vector species, with worldwide representatives (Nault and Ammar 1989). The corn leafhopper *Dalbulus maidis* (De Long and Wolcott) (Hemiptera: Cicadellidae) is widely distributed in tropical areas of the Americas, from southern USA to temperate zones of Argentina (Virla et al. 1990/1991; Giménez Pecci et al. 2002), and it is considered one of the most damaging species to corn due to its role as a vector of maize rayado fino virus, *Spiroplasma kunkelii,* and maize bushy stunt mycoplasm. These three pathogens, alone or in combination, are the ethiological agents of corn stunt, a disease that causes economic losses to corn crops in Mexico and Central and South America, and has been detected in restricted areas in the north of Argentina in 1990 (Giménez Pecci et al. 2000). Among the delphacid pests of maize is the planthopper, *Delphacodes kuscheli* Fennah (Hemiptera: Delphacidae) a native species of Argentina that has been reported as a vector of Mal de Río Cuarto virus (Remes Lenicov et al. 1985; Remes Lenicov and Virla 1999). Due to high incidence and severity of damages, the Mal de Río Cuarto is the most important disease of corn crops in Argentina (Laguna et al. 2000; 2002).

Entomopathogenic fungi are widespread in agroecosystems and belong to a group of microorganisms extensively studied with more than 700 species within 100 genera (Lecuona 1996). These fungi infect a great number of arthropods and hence can be used as pest control agents in an Integrated Pest Management approach (Lecuona 1996). Fungi, particularly some members of Ascomycota, are attractive candidates as biocontrol agents against leafhoppers and planthoppers (Rice and Choo 2000; Toledo et al. 2007), but several studies revealed its failure to invade the insect cuticle, possibly due to the presence of inhibitory compounds such as phenols, quinones, and lipids (Smith and Grula 1981; Szafranek et al. 2001; Howard and Lord 2003; James et al. 2003; Lord and Howard 2004) and also by antibiotic effect of the microbiota living on the cuticular surface of the host (Hubner 1958; Walstad et al. 1970; Schabel 1978). Several works reported antagonistic interactions among microorganisms, such as fungi and bacteria (Currie et al. 1999; Ansari et al. 2005; Alippi and Reynaldi 2006), but there are no studies about the interactions between the microbiota found on the cuticle of hemipterous species and entomopathogenic fungi.

The purpose of the present work was to investigate the interactions among the entomopathogenic fungus *Beauveria bassiana* (Balsamo-Crivelli) Vuillemin (Hypocreales: Cordycipitaceae) and several bacterial antagonists isolated from the cuticular surfaces of *D. maidis* and *D. kuscheli.*

## Materials and Methods

### Insect culture

*Dalbulus maidis* and *Delphacodes kuscheli* were obtained from colonies reared on corn (*Zea mays* L.) and oat (*Avena sativa* L.) respectively in 24 × 9 cm polyethylene terephthalate plastic cages, in a greenhouse at the Facultad de Ciencias Agrarias y Forestales, UNLP (35° S -57° W), La Plata, Buenos Aires, Argentina.

### Fungal isolate and preservation

The *B. bassiana* strain used in this study was isolated from one adult of *Cycloneda sanguinea* L. (Coleoptera: Coccinellidae) associated from corn at El Manantial, Tucumán, Argentina (26° 49′ 50.2″ S - 65° 16′ 59.4″ W). This isolate has been previously reported as pathogenic to planthoppers and leafhoppers (Toledo et al. 2007), and was deposited as ARSEF 8372 in the Collection of Entomopathogenic Fungal Cultures, Agricultural Research Service, Ithaca, New York, USA, and as CEP 147 in the Collection at Centro de Estudios Parasitológicos y de Vectores, La Plata, Buenos Aires, Argentina.

### Isolation and preservation of bacterial strains

Ten adults of each *D. maidis* and *D. kuscheli* (approximately 10d old) were collected from April 2007 to April 2008 at monthly intervals. Using mouth aspirators, insects were placed into glass vials and transported to the laboratory. A total of 120 living insects were evaluated by placing them individually into sterile vials containing 300 μl of sterile distilled water. Each vial was vortex-mixed for 1 min, from which 25 μl were pippeted out and streaked on plates of tryptic soy agar (TSA) (Britania) using a Drigalski spatula. Plates were incubated at 30° C in aerobiosis and examined for bacterial growth every 24 h for and up to 10 days. Potential bacterial antagonists were primarily identified on the basis of Gram reaction and colony morphology. Microscopic examination of bacterial smears stained using the SchaefferFulton technique was made to determine presence and location of spores within cells as well as the size and shape of vegetative cells. The isolates were maintained on sterile mineral water (Glaciar, www.glacierwater.com) at 4° C and stored in tryptic soy broth plus 20 % glycerol (v/v) at 80^°^C.

### Preliminary screening for bacterial strains with antagonistic activity and statistical analysis

A total of 155 bacterial isolates were recovered from the cuticular surface throughout the sampling period, and tested for antagonistic effect against *B. bassiana.* A first screening to evaluate the effect of potential antagonists on the fungal growth was carried out by a central disk test assay (Reynaldi et al. 2004). Briefly, the fungal strain was cultured on malt extract agar for 7 days at 25° C in darkness and a 7 mm mycelium disk from the sporulating area was cut and transferred to the centre of a TSA plate. At the same time, three 7 mm disks containing each bacterial strain from a 48 h culture on TSA were transferred to each plate in the same way and placed at three equidistant points from the central disk. For controls, only a central disk of fungal growth was used. There were 3 replicate plates for each bacterial strain and for each control group (making a total of 65 controls). Treated and control plates were incubated at 30° C and the evaluation was performed by measuring the diameter of the fungal colony at 7 and 10 days, respectively. The percentage of mycelial growth inhibition (MGI) was calculated according to the formula proposed by Michereff et al. (1994).

Only those treatments in which the fungal growth in the presence of bacteria was smaller than that of the controls were included in the statistical analysis. Differences in inhibition growth levels among treatments were analyzed by Kruskal—Wallis test, and means were compared by Fisher's least significant difference (LSD) multiple range test option (*P* ≤ 0.05) using Statgraphics statistical software (STSC, 1994–2001). Bacterial strains isolated from *D. maidis* and *D. kuscheli* were analyzed separately. Differences in biological activity between bacteria isolated from *D. maidis* and those isolated from *D. kuscheli* were analyzed by analysis of variance (ANOVA), and their means were compared by LSD test (*P ≤* 0.05) using the Statgraphics software.

**Table 1.**
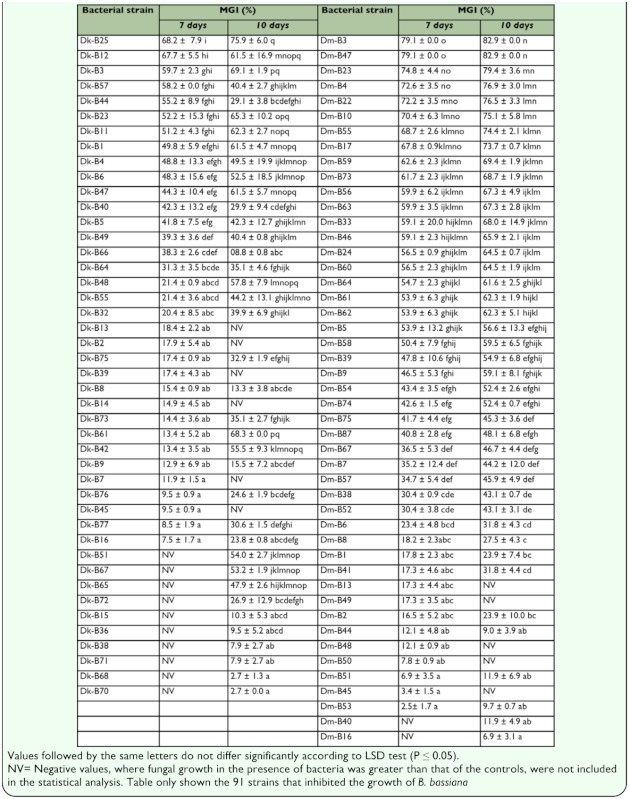
Lifetable, fecundity and rate of natural increase of Commom Hoopae louse (*Upupicola upupae*).

### Identification of selected bacterial antagonists

Twenty-four bacterial isolates that showed the most effective antagonist effect against *B. bassiana* (MGI values between 40% and 83%) were further characterized to identify them at species level. Tests performed include catalase reaction, oxidase activity, motility, lipid globule staining, production of lecithinase, haemolytic activity, reduction of nitrate, anaerobic utilization of glucose, mannitol and arabinose utilization, and starch and gelatin hydrolysis according to standard protocols (Gordon et al. 1973). When necessary, API 20E and API 50CH strips plus API 50CHB medium and data base Apiweb (Biomerieux, www.biomerieux.com) were used.

**Figure 1.**
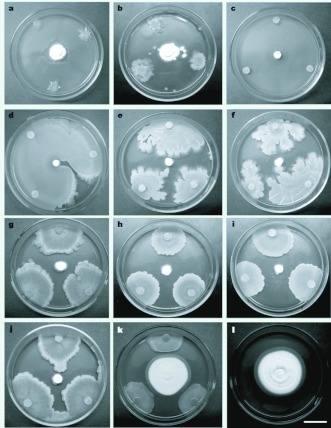
Antifungal activity of *Bacillus licheniformis* Dk-B23 (plate a), *B*. *pumilus* Dk-B12, Dk-B25, Dm-B3, Dm-B22, Dm-B23 (plates b, c, d, e and f), and *B*. *subtilis* Dk-B57, Dm-B4, Dm-Bl7, Dm-B55 (plates g, h, i and j) against *B*. *bassiana* on TSA plates after 7 days post-incubation. Controls: bacterial strain that did not exhibit antifungal activity (plate k) and fungal growth without bacteria after 7 d of incubation (plate 1). Scale bar: 1.8 cm. High quality figures are available online.

### Antagonistic activity against conidial germination of *B. bassiana*

According to the results obtained in the preliminary screening, ten bacterial strains were selected for testing their antagonistic activity on the conidial germination of *B. bassiana* ARSEF 8372 by means of a paired suspension assay. *B. bassiana* was cultured on malt extract agar and incubated for 8 days at 26° C in darkness. Conidia were harvested with a sterile loop and placed into test tubes containing 5 ml of Tween 80 (0.1 % v/v) (Sigma, www.sigmaaldrich.com). The suspensions were vortex-mixed for 1 min, filtered through a sterile muslin layer, and adjusted to a concentration of 1 × 10^8^ conidia/ml after determination of conidial concentration using a Neubauer hemacytometer. Each test bacterium suspension (vegetative cells after 24 h incubation or spores after 7 days incubation on TSA) was adjusted to a concentration of 0.5 Mc Farland. Bacterial suspensions were prepared in Tween 80 (0.1 % v/v).

Five μl of each conidial suspension and 5 μl of each bacterial suspension (vegetative cells or spores) were deposited on the surface of a microscopic slide containing 100 μl of water agar medium as a substrate. The slides were placed over moist filter paper inside sterile 90 mm-diameter Petri dishes and incubated in darkness at 30° C. After 24 h germinated conidia were counted under a light microscope (400 X) by counting 3 times 100 conidia for each fungus-bacterial combination and each control taking into account that germinated conidia are those exhibiting a germ tube greater than the conidial diameter (usually once or twice). There were 3 replicates and one control per treatment. The whole assay was run twice over time in the same conditions mentioned above. The differences in inhibition growth levels among treatments were analyzed by Kruskal—Wallis analysis, and means were separated by LSD test (P ≤ 0.05) using Statgraphics statistical software. In addition, the abnormalities of the conidial germ tubes, if any, were registered.

## Results

### Inhibition of mycelial growth

A total of 155 bacterial isolates were obtained from the cuticular surface of *D. maidis* and *D. kuscheli.* Eighty-three isolates collected from *D. maidis* were represented by 52% Grampositive spore-forming bacilli, 37% Gram-positive non-spore-forming bacilli, 4% Gram-negative bacilli, and 7% Gram-positive cocci, whereas the 72 isolates from *D. kuscheli* were represented by 46% Gram-positive sporeforming bacilli, 22% Gram-positive nonspore-forming bacilli, 6% Gram-negative bacilli, and 26% Gram-positive cocci. As shown in Table 1,91 out of 155 strains tested inhibited the growth of *B. bassiana.* After 7 days of incubation significant differences among treatments were recorded for *D. kuscheli* (*K*= 81.9; *P*= 0.00). Strains Dk-B3, Dk-B11, Dk-B12, Dk-B23, Dk-B25, Dk-B44, and Dk-B57 showed the most effective antagonistic effect against *B. bassiana,* with percentages of MGI of 50% or more. After 10 days of incubation significant differences were also observed among treatments (*K=* 89.3; *P=* 0.00), the most effective antagonists were Dk-B1, Dk-B3, Dk-B6, Dk-B11, DkB12, Dk-B23, Dk-B25, Dk-B42, Dk-B47, DkB48, Dk-B51, Dk-B61, and Dk-B67. In relation to *D. maidis,* significant differences were also recorded among the 83 strains isolates tested after 7 d (*K* = 118.2; *P* = 0.00) and 10 d (*K* = 108.0; *P* = 0.00) after incubation. Strains Dm-B3, Dm-B4, Dm-B5, Dm-B10, Dm-B 17, Dm-B22, Dm-B23, DmB24, Dm-B33, Dm-B46, Dm-B47, Dm-B55, Dm-B56, Dm-B58, Dm-B59, Dm-B60, DmB61, Dm-B62, Dm-B63, Dm-B64, and DmB73 were the most effective antagonists (Table 1 and Figure 1).

**Table 2.**
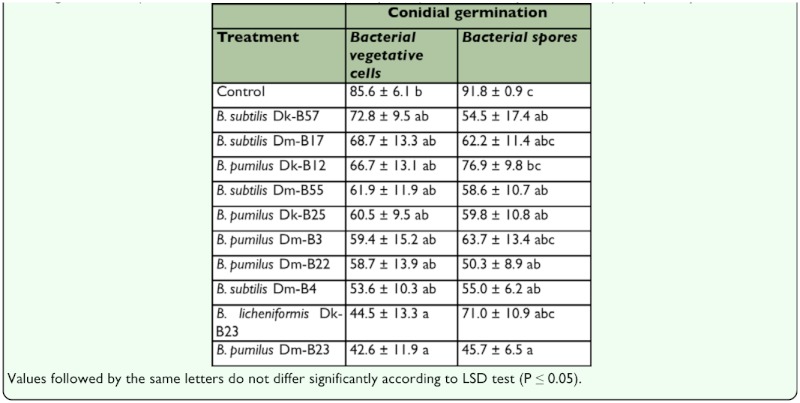
Inhibition of *B*. *bassiana* conidial germination (% mean ±SE) by selected bacterial antagonists preparations obtained from vegetative cells (cutures of 24 hours of incubation) or spores (cultures of 7 days of incubation), respectively.

More bacterial strains isolated from *D. maidis* were more antagonistic to *B. bassiana* than those from *D. kuscheli* at 7 days (*F* = 5.76; df = 1; 77; *P =* 0.018) and 10 days (*F* = 8.16; df = 1; 78; *P* = 0.0055) of incubation, respectively. Twenty-five isolates from *D. maidis* and 13 isolates from *D. kuscheli* showed values of MGI of 50% or more, respectively (Table 1 and Figure 1).

**Table 3.**
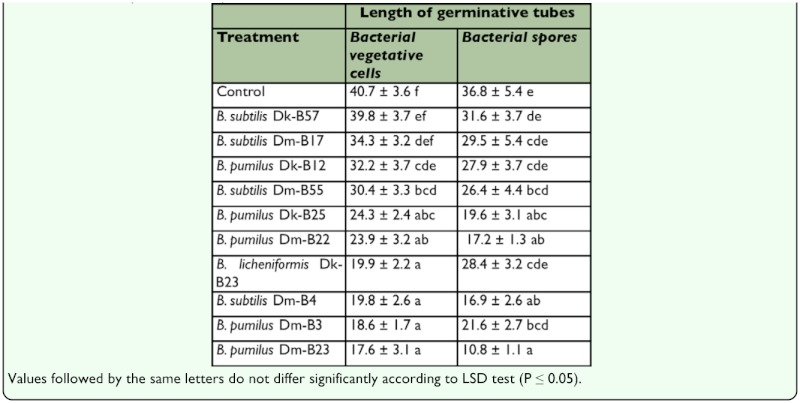
Effects of bacterial antagonist preparations obtained from vegetative cells or spores, upon the growth of germinative tubes of *B. bassiana* (pm mean ± SE).

Among the most effective antagonistic strains, Dk-B1, Dk-B11, Dm-B9, Dm-B24, Dm-B54, and Dm-B58 were identified as *Bacillus thuringiensis* Berliner (Bacillales: Bacillaceae), Dk-B3 as *Bacillus mycoides* Flügge, Dm-B5, Dm-B10, Dm-B47, Dm-B59, Dm-B60, Dm-B61, Dm-B62, and Dm-B63 as *Bacillus megaterium* de Bary. Using the API 20E and API 50CH strips and data base Api*web* isolates Dk-B12, Dk-B25, Dm-B3, Dm-B22, and Dm-B23 matched as *Bacillus pumilus* Meyer and Gottheil (99.9%, 99.4%, 99.9%, 99.9%, and 99.5% ID, respectively). Strain Dk-B23 matched as *Bacillus licheniformis* (Weigmann) Chester (97.3% ID) and Dk-B57, Dm-B4, Dm-B 17, and Dm-B55 matched as *Bacillus subtilis* (Ehrenberg) Cohn (85.3%, 98.1%, 99,1%, and 70.9% ID, respectively).

### Inhibition of conidial germination

After 24 h of incubation no significant differences were found among treatments for vegetative cells (*K*= 13.4; *P*= 0.2) or spores (*K*= 16.3; *P*= 0.1), although *B. pumilus* DmB23 showed a higher inhibitory activity in both cases (Table 2). In addition, significant differences in conidial germ tube lengths were found for both vegetative cells (*K*= 88.3; *P*= 0.00) and spores (*K*= 45.4; *P*= 0.00), with *B. pumilus* Dm-B23 found to be the most effective antagonist (Table 3). No abnormalities of the conidial germ tubes were found in any of the bacterial-fungus combinations tested.

## Discussion

According to the results presented here, Gram-positive aerobic spore forming bacteria belonging to the species *B. megaterium, B. mycoides, B. pumilus, B. licheniformis, B. subtilis,* and *B. thuringiensis* showed the most effective antagonistic effect on *B. bassiana* mycelial growth and conidial germination.

*Bacillus pumilus, B. licheniformis,* and *B. subtilis* along with *Bacillus atrophaeus* and *B. amyloliquefaciens* are closely related species that comprise the *Bacillus subtilis* group (Wattiau et al. 2001). The ability of this group of bacteria to inhibit fungal and bacterial growth by secreting antibiotics, antibiotic-like compounds, bacteriocins, or antifungal compounds has been well documented (Thimon et al. 1992; Feignier et al. 1995; Leifert et al. 1995; Gálvez et al. 1993; Martinari et al. 2002). These substances could play an important role in antagonistic interactions between microorganisms, which may be based on parasitism, direct competition, or antibiosis (Singh and Faull 1988).

Bacterial strains isolated from *D. maidis* were significantly more antagonistic, or at least produced large amounts of antagonistic compounds against *B. bassiana* than those isolates from *D. kuscheli.* The failure of *B. bassiana* to invade the *D. maidis* cuticle and the greater mortality rates previously observed in *D. kuscheli* could be related to a less antagonistic activity of the bacteria living in the same ecological niche. This hypothesis might explain those results observed in previous studies (Toledo et al. 2007) reporting that *D. maidis* mortality caused by *B. bassiana* was 23% less than that of *D. kuscheli* after 14 d post-inoculation. Although, further bioassays will be necessary to clarify this hypothesis by testing insects previously treated with antibiotics and inoculated separately with each bacterial strain and then with the pathogenic fungus.

This is the first report of bacterial isolates obtained from cuticular surfaces of Cicadellids and Delphacids able to inhibit the growth of the entomopathogenic fungus *B. bassiana.* Other examples of bacterial strains having antifungal activity include strains of *B. pumilus* against Mucoraceae, *Aspergillus flavus,* and *A. parasiticus* (Eurotiales: Trichocomaceae) species (Bottone and Peluso 2003; Cho et al. 2009) and also against *Bipolaris sorokiniana* (Pleosporales: Pleosporaceae) and *Septoria tritici* (Capnodiales: Mycosphaerelaceae) (Alippi et al. 2000) have been reported. In addition, antagonistic effects of *B. subtilis* strains against different phytopathogenic fungi like *Colletotrichum trifolii* (Phyllachorales: Phyllachoraceae) (Douville and Boland 1992), *Exserohilum turcicum* (Pleosporales: Pleosporaceae) (Reis et al. 1994), *A. flavus* (Moyne et al. 2001), *Pythium aphanidermatum* (Pythiales: Pythiaceae) (Leclere et al. 2005), *B. sorokiniana, S. tritici,* and *Alternaria triticimaculans* (Dothideales: Pleosporaceae) (Alippi et al. 2000) and also against entomopathogenic fungi like *Ascophaera apis* (Eurotiales: Ascophaeraceae), the causative agent of chalkbrood disease in honeybee larvae (Basim and Gürel 1999; Reynaldi et al. 2004) have been established. Additionally *B. licheniformis* strains with antifungal compounds against a wide variety of plant pathogenic fungi have been isolated (Galvez et al. 1993; Lebbadi et al. 1994; Alippi et al. 2000). Similar results have been reported for *B. megaterium* on *A. apis* (Reynaldi et al. 2004; Gilliam 1993), on *S. tritici* (Kildea et al. 2008), and on *Phytophtora capsici* (Peronosporales: Pythiaceae) (Akgul and Mirik 2008). It is interesting to point out that previous reports showed that symbiotic bacteria of entomopathogenic nematodes as *Xenorhabdus nematophilus, X. bovienii,* and *Photorhabdus luminescens* were antagonistic to the entomopathogenic fungi *B. bassiana* and *M. anisopliae* (Hypocreales: Clavicipitaceae) by inhibiting their growth and conidial production (Barbercheck and Kaya 1990; Chen et al. 1994; Ansari et al. 2005).

Our findings suggest the existence of a kind of antimicrobial activity possibly due to antibiosis effect and/or direct competition of spore-forming bacteria associated with Cicadellidae and Delphacidae that can reduce or inhibit the growth of *B. bassiana.* The presence of bacteria belonging to *Bacillus cereus* sensu lato group, *B. megaterium, B. subtilis* and closely related species in the cuticle of hemipterous insects could be an obstacle for the optimization and promotion of the use of entomopathogenic fungi in an integrated pest management approach in corn crops. Further studies are needed in order to clarify these microbial interactions and to characterize the chemical nature of the compounds involved in the inhibitory activities.

## Abbreviations:

**LSD**,: least significant difference,
MGI,: mycelial growth inhibition,
TSA,: tryptic soy agar
